# Selecting Tumor-Specific Molecular Targets in Pancreatic Adenocarcinoma: Paving the Way for Image-Guided Pancreatic Surgery

**DOI:** 10.1007/s11307-016-0959-4

**Published:** 2016-04-29

**Authors:** Susanna W. L. de Geus, Leonora S. F. Boogerd, Rutger-Jan Swijnenburg, J. Sven D. Mieog, Willemieke S. F. J. Tummers, Hendrica A. J. M. Prevoo, Cornelis F. M. Sier, Hans Morreau, Bert A. Bonsing, Cornelis J. H. van de Velde, Alexander L. Vahrmeijer, Peter J. K. Kuppen

**Affiliations:** 1 Department of Surgery, Leiden University Medical Center, Albinusdreef 2, 2300 RC Leiden, The Netherlands; 2 Department of Pathology, Leiden University Medical Center, Leiden, The Netherlands

**Keywords:** Pancreatic adenocarcinoma, Periampullary adenocarcinoma, Molecular imaging, Image-guided surgery, Immunohistochemistry, Integrin α_v_β_6_, Carcinoembryonic antigen (CEA), Epithelial growth factor receptor (EGFR), Urokinase plasminogen activator receptor (uPAR)

## Abstract

**Purpose:**

The purpose of this study was to identify suitable molecular targets for tumor-specific imaging of pancreatic adenocarcinoma.

**Procedures:**

The expression of eight potential imaging targets was assessed by the target selection criteria (TASC)—score and immunohistochemical analysis in normal pancreatic tissue (*n* = 9), pancreatic (*n* = 137), and periampullary (*n* = 28) adenocarcinoma.

**Results:**

Integrin α_v_β_6_, carcinoembryonic antigen (CEA), epithelial growth factor receptor (EGFR), and urokinase plasminogen activator receptor (uPAR) showed a significantly higher (all *p* < 0.001) expression in pancreatic adenocarcinoma compared to normal pancreatic tissue and were confirmed by the TASC score as promising imaging targets. Furthermore, these biomarkers were expressed in respectively 88 %, 71 %, 69 %, and 67 % of the pancreatic adenocarcinoma patients.

**Conclusions:**

The results of this study show that integrin α_v_β_6_, CEA, EGFR, and uPAR are suitable targets for tumor-specific imaging of pancreatic adenocarcinoma.

## Introduction

Pancreatic adenocarcinoma currently ranks the fourth leading cause of cancer-related death in the Western world, with a 5-year survival rate of less than 5 % [1]. Radical surgical tumor resection is imperative to curative treatment of these patients as positive resection margins (defined as tumor cells present at the surface of the resection margins of the surgical specimen) are associated with a dramatic decrease in median overall survival [1–4]. Unfortunately, positive resection margins are common after pancreatic surgery and reported rates vary between 24 % and 76 % [5–7]. Adjuvant therapy cannot retaliate the poor survival outcome associated with residual disease [8]. The disappointing irradical resection rates after pancreatic surgery are due to our current inability to detect the true delineation of the tumor extent during surgery, which is further complicated by the intricate anatomy of the pancreas and the commonly present peritumoral inflammatory zone in pancreatic cancer. Conventional anatomic imaging modalities used for preoperative diagnosis, staging, and surgical planning include multiphase intravenous contrast-directed thin slice computed tomography, magnetic resonance imaging, endoscopic ultrasonography, and endoscopic retrograde cholangiopancreatography [9, 10]. However, the translation of these preoperative imaging techniques to the surgical field remains challenging and in the theater, the surgical oncologist solely has to rely on vision and manual palpation to discriminate between malignant and healthy pancreatic tissue, assisted by ultrasonography and pathologic evaluation of frozen tissue sections [10].

Intraoperative tumor-specific imaging offers the opportunity to significantly improve current practice by increasing the capability to obtain negative resection margins and visualize residual disease during pancreatic surgery. This novel imaging approach uses labeled receptor ligands, nanoparticles, antibodies, or antibody fragments targeting cancer-specific antigens on the tumor surface detected by positron emission tomography, single-photon emission computed tomography, ultrasonography, magnetic resonance, and/or near-infrared fluorescence imaging modalities [11–13]. The feasibility of these imaging techniques has already successfully been proven in glioma and ovarian cancer surgery using respectively the fluorescent agents 5-aminolevulinic acid and folate conjugated to fluorescein isothiocyanate [11, 14]. Furthermore, the potential of image-guided surgery in pancreatic adenocarcinoma has been demonstrated by numerous preclinical studies using cancer-specific contrast agents targeting integrin α_v_β_6_, carcinoembryonic antigen (CEA), epithelial growth factor receptor (EGFR), human epidermal growth factor receptor (HER2), urokinase plasminogen activator receptor (uPAR), or vascular endothelial growth factor receptor 2 (VEGFR2) among others (Table 1). Nevertheless, the orthotopic mouse models used in these studies are based on a small number of pancreatic adenocarcinoma cell lines originating from single patients and therefore less representative for the potential of these imaging probes in the overall population of pancreatic cancer patients. The translation from bench to bedside of this promising imaging strategy for pancreatic adenocarcinoma currently hinges on the lack of tumor-specific and thoroughly evaluated molecular targets expressed on the general population of pancreatic adenocarcinoma patients for the further development of tumor-targeting contrast agents [15, 16].

**Table 1:** Overview of the characteristics and preclinical experience with tumor-specific imaging of integrin α_v_β_6_, carcinoembryonic antigen (CEA), hepatocyte growth factor receptor (cMET), epithelial growth factor receptor (EGFR), epithelial cell adhesion molecule (EpCAM), human epidermal growth factor receptor (HER2), urokinase plasminogen activator receptor (uPAR), and vascular endothelial growth factor receptor 2 (VEGFR2) in pancreatic adenocarcinoma animal models

Target	Type of receptor (family)	Function	Tumor-specific probe		Imaging modality	Pancreatic cancer xenograft	Ref.
Integrin α_v_β_6_	Transmembrane receptor (integrin family of cell adhesion receptors) [63]	Controls extracellular matrix remodeling and provides the traction necessary for cell motility. Tumor cell migration, invasion, and proliferation [63]	Peptide	^18^F-fluorobenzoic acid	PET	BxPC-3	[64, 65]
			Peptide	^99m^TC	SPECT/CT	BxPC-3	[66]
			Peptide	Phthalocyanine dye	NIRF imaging	BxPC-3	[67]
			Peptide	^18^F-fluorobenzoate	PET	BxPC-3	[68]
CEA	Glycoprotein (immunoglobulin superfamily) [69]	Tumor cell migration, circulation, implantation and proliferation, which is facilitated by the immunosuppressive effect of CEA [70]	scFv	800CW	NIRF imaging	BxPC-3	[71]
			MAB	IR700	NIRF imaging	BxPC-3	[72]
			MAB	AlexaFluor 488	NIRF imaging	BxPC-3	[73–77]
			scFv	I^124^	PET/CT	BxPC-3	[78]
cMET	Tyrosine kinase receptor (HGFR family) [79]	Tumor cell proliferation, survival, motility, and invasion [79]	–	–	–	–	–
EGFR	Tyrosine kinase receptor (ErbB family) [80]	Induces tumor cell differentiation and proliferation [81]	F(ab’)_2_ fragments	^64^Cu	PET/CT	PANC-1	[82]
			MAB	CF-750	MSOT	MiaPaCa-2	[83]
			scFv	IONP	MRI	MiaPaCa-2	[84, 85]
			XIMAB	^86^Y	PET	SHAW	[86]
EpCAM	Transmembrane glycoprotein [87]	Tumor cell proliferation, migration, and mitogenic signal transduction [87]	–	–	–	–	–
HER2	Tyrosine kinase receptor (ErbB family) [88]	Tumor cell proliferation, survival, adhesion, and migration [88]	MAB	^111^In	PET	PC-Sw	[89]
uPAR	GPI-anchored receptor (plasminogen activation system) [71]	Tumor cell migration, proliferation, and survival [90]	ATF-uPA	NIR-830, IONP	NIRF imaging, MRI	MiaPaCa-2	[84, 91–93]
			MAB	Cy5.5	NIRF imaging	AsPC-1	[94]
VEGFR2	Tyrosine kinases receptor (VEGFR family) [95]	Angiogenesis during tumorgenesis [95]	MAB	Microbubbles	US	Transgenic mouse model	[96–98]

*ATF* amino terminal fragement, *CT* computed tomography, *FDA* Food and Drug Administration, *HGFR* hepatocyte growth factor receptor, *MAB* monoclonal antibody, *MPIO* microparticles of iron oxide, *MSOT* multispectral optoacoustic tomography *NIRF* near-infrared fluorescence, *NPIO* nanoparticles of iron oxide, *PC* pancreatic cancer, *PET* positron emission tomography, *scFv* single-chain antibody fragments, *SPECT* single-photon emission computed tomography, *uPA* urokinase plasminogen activator, *US* ultrasound, *VEGF* vascular endothelial growth factor, *VEGFR* vascular endothelial growth factor receptor, *XIMAB* chimeric human-mouse antibodies

Therefore, the aim of this study was to explore the suitability of integrin α_v_β_6_, CEA, hepatocyte growth factor receptor (cMET), EGFR, epithelial cell adhesion molecule (EpCAM), HER2, uPAR, and VEGFR2 as molecular targets for tumor-targeted imaging of pancreatic adenocarcinoma patients. The primary endpoint of this study was to evaluate the ability of these markers to distinguish between normal pancreatic tissue and pancreatic and periampullary adenocarcinoma by performing immunohistochemistry on surgical specimen of these malignancies and normal pancreatic tissue obtained adjacent to the tumor. In addition, these biomarkers were judged on the Target Selection Criteria (TASC) proposed by Van Oosten et al. [17].

## Materials and Methods

### Patient Selection

Medical records and pathology specimens of 137 patients with pancreatic ductal adenocarcinoma and 28 patients with periampullary adenocarcinoma who underwent pancreatic surgery at Leiden University Medical Center (LUMC) between June 2002 and July 2012 were retrospectively reviewed. Periampullary adenocarcinoma were included to assess the potential of tumor-specific imaging targets to visualize every pancreatic head mass, since preoperative differentiation between pancreatic, distal bile duct, ampullary, and duodenal adenocarcinoma can be challenging [18]. For the purpose of this study, periampullary adenocarcinoma was defined as adenocarcinoma that invades the pancreas arising from the ampulla of Vater, duodenum, or distal bile duct [19]. Patients who received any form of neoadjuvant chemotherapy and/or radiotherapy were excluded from this study, since this may influence the expression of molecular markers [20]. In addition, normal pancreatic tissue adjacent to the tumor was also obtained from nine patients to evaluate the tumor specificity of the biomarkers. Clinicopathological data from these patients were retrospectively collected from electronic hospital records. Tumor differentiation grade was determined according to the guideline of the World Health Organization, and the TNM stage was defined according to the American Joint Commission on Cancer criteria [21]. All samples were nonidentifiable and used in accordance with the ethical standards of the institutional research committee and with the 1964 Helsinki declaration and its later amendments.

### Immunohistochemistry

Tissue microarrays (TMAs) of tumor and normal tissues were constructed to perform uniform and simultaneous immunohistochemical stainings to limit intra-assay variations. Formalin-fixed paraffin-embedded tissue blocks of the primary tumor were collected from the archives of the Pathology Department. A single representative block was selected for each patient based on hematoxylin-eosin-stained sections. From each donor block, triplicate 2.0-mm cores were punched from areas with clear histopathological tumor representation and transferred to a recipient TMA block using the TMA Master (3DHISTECH, Budapest, Hungary). From each completed TMA block and normal pancreatic tissue block, 5-μm sections were sliced. The sections were deparaffined in xylene and rehydrated in serially diluted alcohol solutions, followed by demineralized water according to standard protocols. Endogenous peroxidase was blocked by incubation in 0.3 % hydrogen peroxide in phosphate-buffered saline (PBS) for 20 min. For EpCAM, c-MET, HER2, and uPAR staining antigen retrieval was performed by heat induction at 95 °C using PT Link (Dako, Glostrup, Denmark) with a low-pH Envision FLEX target retrieval solution (citrate buffer pH 6.0, Dako). VEGFR staining required antigen retrieval with high-pH Envision FLEX target retrieval solution (Tris-EDTA pH 9.0, Dako). For staining of EGFR and integrin α_v_β_6_, antigen retrieval was performed with 0.4 % pepsin incubation for 10 min at 37 °C. CEA staining did not require antigen retrieval. Immunohistochemical staining was performed by incubating tissue microarrays overnight with antibodies against VEGFR2 (55B11; Cell Signaling Technology, Danvers, MA, USA), EpCAM (323A3, in-house produced hybridoma), c-MET (SC10; Santa Cruz Biotechnology, Santa Cruz, CA, USA), CEA (A0155; Dako, Glustrup, Denmark), EGFR (E30; Dako), integrin α_v_β_6_ (6.2A; Biogen Idec MA Inc., Cambridge, MA, USA), HER2 (A0485; Dako), and uPAR (ATN-615, kindly provided by Prof A.P. Mazar, Northwestern University, Evanston, IL) all at room temperature [22, 23]. All antibodies were used at predetermined optimal dilutions using proper positive and negative control tissue. Furthermore, all antibodies selected for this study were solely selective for integrin α_v_β_6_, CEA, cMET, EGFR, EpCAM, HER2, uPAR, and VEGFR respectively, except for the CEA antibody (A0155; Dako) that was also sensitive to CEA-like proteins (CEACAM1, CEACAM3, CEACAM4, CEACAM 6, CEACAM7, CEACAM 8) and the uPAR antibody (ATN-615) that also recognizes the soluble form of uPAR suPAR [22]. Negative control samples were incubated with PBS instead of the primary antibodies. The sections were washed with PBS, followed by incubation with Envision anti-mouse (K4001; Dako) or Envision anti-Rabbit (K4003; Dako), where applicable, for 30 min at room temperature. After additional washing, immunohistochemical staining was visualized using 3,3-diaminobenzidine tertahydrochloride solution (Dako) for 5–10 min resulting in brown color and counterstained with hematoxylin, dehydrated, and finally mounted in pertex. All stained sections were scanned and viewed at ×40 magnification using the Philips Ultra Fast Scanner 1.6 RA (Philips, Eindhoven, Netherlands). The numerical value for overall intensity (intensity score) was based on a four-point system: 0, 1, 2, and 3 (for none, light, medium, or high intense staining), as previously described by Choudhury et al., and staining was considered positive if >10 % of the tumor cells expressed a medium or dark staining pattern [23–29]. Evaluation of the immunohistochemical staining of all molecular targets was performed blinded and independently by two observers (S.W.L.G. and H.A.J.M.P). In case of disagreement, the stainings were discussed until agreement was reached.

### Target Selection Criteria

The TASC score is based on granting points for the following seven characteristics of suitable molecular targets: extracellular protein localization (receptor bound to cell surface, 5 points; in close proximity of the tumor cell, 3 points); diffuse upregulation through tumor tissue (4 points); tumor-to-healthy cell (T/N) ratio (T/N ratio >10, 3 points); high percentage upregulation in patients (>90 %, 6 points; 70–90 %, 5 points; 50–69 %, 3 points; 10–49 %, 0 points); previous imaging success *in vivo* (2 points); enzymatic activity (1 point); and target-mediated internalization (1 point). All biomarkers were granted points for the seven characteristics and a total score of 18 or higher indicated that the biomarker is potentially suitable for tumor-targeted imaging *in vivo* [17]. Whereas a T/N ratio could not be obtained from immunohistochemical staining, we simplified the T/N ratio to a significant lower staining intensity in normal pancreatic tissue compared to pancreatic and periampullary adenocarcinoma. For the purpose of this study, diffuse expression was defined as staining in ≥50 % of tumor cells in the majority (>50 %) of the patients; focal expression as staining in <50 % of tumor cells in the majority (>50 %) of the patients and negative expression as staining in 0 % of the tumor cells in the majority (>50 %) of the patients.

### Statistical Analysis

The statistical analysis was performed using SPSS version 23.0 software (SPSS, © IBM Corporation, Somer NY, USA) and GraphPad Prism 6 (GraphPad, Software, Inc., La Jolla, CA, USA). Interobserver variation of immunohistochemical results was analyzed using Cohen’s kappa coefficient, and >0.8 was considered as acceptable. Baseline characteristics between groups were analyzed using chi-squared test for categorical data. Immunohistochemistry staining intensity in normal pancreatic tissue was compared to pancreatic and periampullary adenocarcinoma using the independent Student’s *t* test. In all tests, results were considered statistically significant at the level of *p* < 0.05.

## Results

### Patient and Tumor Characteristics

In total, 165 patients were included, whereof 137 and 28 with pancreatic and periampullary adenocarcinoma, respectively (Table 2). The mean age was 66 years and ranged between 38 and 84 years. Most tumors were T-stage 3 (50.9 %) and poorly differentiated (44.6 %). Regional lymph node involvement was found in 69.7 % of patients. The majority of the patients received no adjuvant therapy after surgery. Patients diagnosed with adenocarcinoma originating from the pancreas had, compared to patients diagnosed with periampullary adenocarcinoma, more frequently lymph node invasion (75 vs. 43 %; *p* < 0.001), positive surgical margins (31 vs. 11 %; *p* = 0.037), vascular invasion (33 vs. 11 %; *p* = 0.023), perineural invasion (64 vs. 37 %; *p* = 0.011), and received more often adjuvant therapy (50 vs. 7 %; *p* < 0.001).

**Table 2:** Baseline characteristics for the patients with pancreatic and periampullary adenocarcinoma included in this study

Characteristics	Total population (n = 165)	Pancreatic adenocarcinoma (n = 137)	Periampullary adenocarcinoma (n = 28)	p-value
Age, *n* (%)				
<65 years	76 (46.1 %)	66 (48.2 %)	10 (35.6 %)	0.228
≥65 years	89 (53.9 %)	71 (51.8 %)	18 (64.3 %)	
Gender, *n* (%)				
Male	80 (48.5 %)	66 (48.2 %)	14 (50.0 %)	0.860
Female	85 (51.5 %)	71 (51.8 %)	14 (50.0 %)	
Tumor location, *n* (%)				
Pancreatic head	155 (93.9 %)	127 (92.7 %)	28 (100.0 %)	–
Other	10 (6.1 %)	10 (7.3 %)	–	
Tumor differentiation, *n* (%)				
Well differentiated	17 (13.3 %)	12 (8.8 %)	5 (17.9 %)	0.224
Moderately differentiated	54 (42.2 %)	43 (31.4 %)	11 (39.3 %)	
Poorly/undifferentiated	57 (44.6 %)	45 (32.8 %)	12 (42.8 %)	
Missing	37	37	–	
Tumor size, *n* (%)				
<30 mm	97 (59.9 %)	77 (57.5 %)	20 (71.4 %)	0.170
≥30 mm	65 (40.1 %)	57 (42.5 %)	8 (28.6 %)	
Missing	3	3	–	
Primary tumor, *n* (%)				
pT1	31 (18.8 %)	21 (15.3 %)	10 (35.7 %)	0.071
pT2	40 (24.2 %)	36 (26.3 %)	4 (14.3 %)	
pT3	84 (50.9 %)	72 (52.6 %)	12 (42.9 %)	
pT4	10 (6.1 %)	8 (5.8 %)	2 (7.1 %)	
Regional lymph node, *n* (%)				
pN0	50 (30.3 %)	34 (24.8 %)	16 (57.1 %)	<0.001
pN1	115 (69.7 %)	103 (75.2 %)	12 (42.9 %)	
Surgical margin status, *n* (%)				
R0	119 (72.6 %)	95 (69.3 %)	24 (88.9 %)	0.037
R1	45 (27.4 %)	42 (30.7 %)	3 (11.1 %)	
Adjuvant therapy, *n* (%)				
Yes	70 (42.4 %)	68 (49.6 %)	2 (7.1 %)	<0.001
No	95 (57.6 %)	69 (50.4 %)	26 (92.9 %)	
Vascular invasion, *n* (%)				
Positive	48 (29.3 %)	45 (32.8 %)	3 (11.1 %)	0.023
Negative	116 (70.7 %)	92 (67.2 %)	24 (88.9 %)	
Perineural invasion, *n* (%)				
Positive	97 (59.1 %)	87 (63.5 %)	10 (37.0 %)	0.011
Negative	67 (40.9 %)	50 (36.5 %)	17 (63.0 %)	

*p Value was obtained for patients with pancreatic adenocarcinoma compared to periampullary adenocarcinoma patients, and *p* < 0.05 was considered significant

### Biomarker Expression

**Fig. 1:**
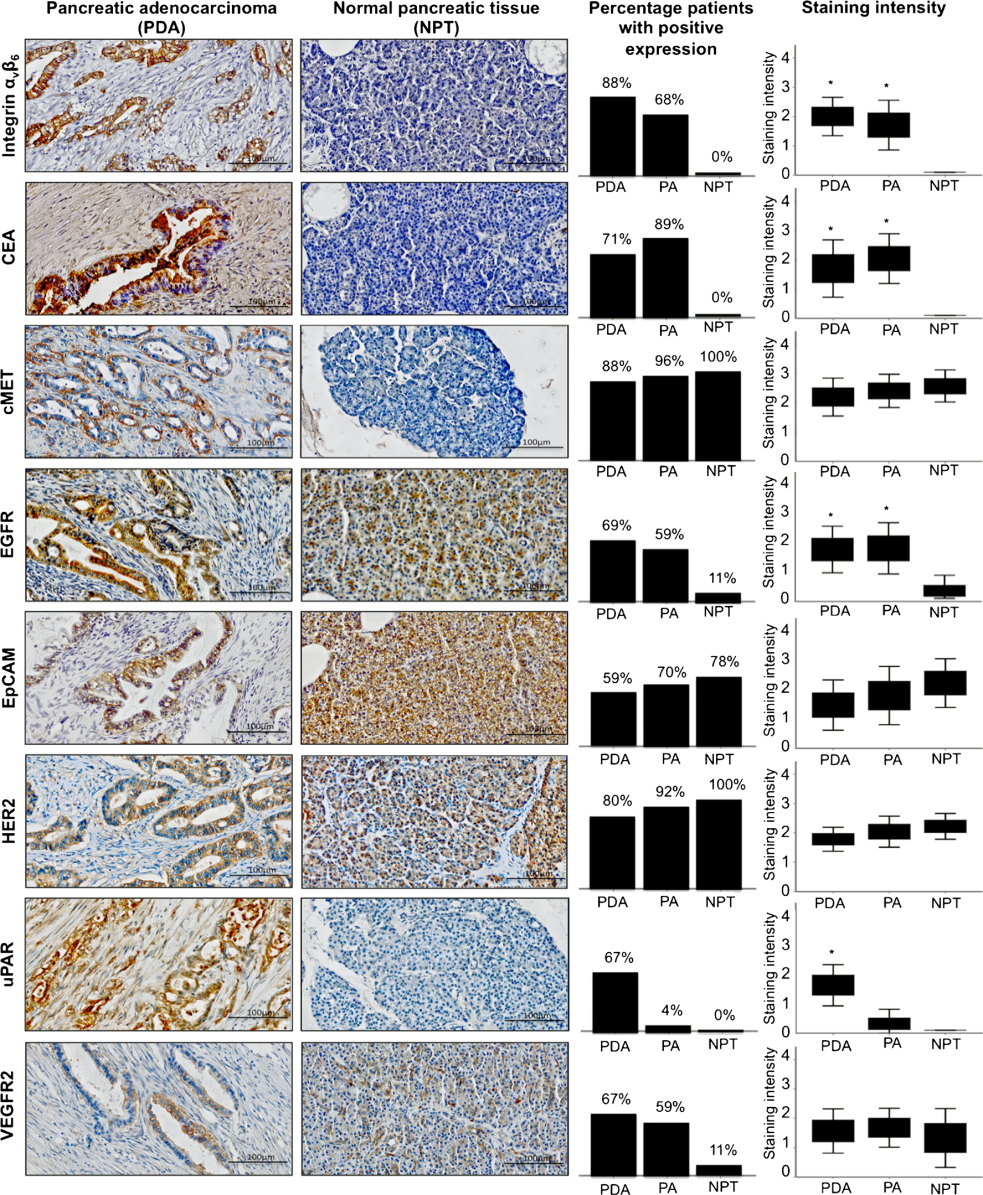
Representative images of moderate immunohistochemistry staining in pancreatic adenocarcinoma (*left column*) and absent or present immunohistochemistry expression in pancreatic adenocarcinoma (*second left column*), followed by bar charts (*third left column*) displaying the percentage of PAC patients with positive staining (positive staining was defined as moderate or strong expression in >10 % of tumor cells) and boxplots (*right column*) showing the mean immunohistochemistry staining (staining intensity was classified for every patient as followed: 0 = negative, 1 = weak, 2 = moderate, and 3 = strong) in pancreatic adenocarcinoma (PDA), periampullary adenocarcinoma (PA), and normal pancreatic tissue (NPT) for integrin α_v_β_6_, carcinoembryonic antigen (CEA), hepatocyte growth factor receptor (cMET), epithelial growth factor receptor (EGFR), epithelial cell adhesion molecule (EpCAM), human epithelial growth factor receptor (HER2), urokinase receptor (uPAR), and vascular endothelial growth factor receptor 2 (VEGFR2) expression. *Significant difference in staining intensity (defined as *p* value of 0.05) in pancreatic or periampullary adenocarcinoma compared to normal pancreatic tissue.

Of the 165 pancreatic and periampullary adenocarcinoma specimens collectively present on the TMA, 159 specimens (96 %) could successfully be microscopically quantified for integrin α_v_β_6_ expression, 158 (96 %) for CEA, 159 (96 %) for cMET, 156 (95 %) for EGFR, 151 (92 %) for EpCAM, 152 (92 %) for HER2, 155 (94 %) for VEGFR2, and 152 (92 %) for uPAR. The missing cases were due to staining artifacts, excessive necrotic tissue, or unacceptable tissue loss during the staining procedure. The molecular markers showed mainly membranous and cytoplasmic immunoreactivity in pancreatic and periampullairy adenocarcinoma cells; CEA and uPAR also showed stromal immunoreactivity (Fig. 1). Diffuse membranous staining was found for integrin α_v_β_6_, CEA, cMET, EGFR, HER2, and uPAR in pancreatic adenocarcinoma (Table 3) and integrin α_v_β_6_, CEA, cMET, EGFR, EpCAM, HER2, and VEGFR2 in periampullary adenocarcinoma (Table 4). Immunohistochemistry staining, if present, in healthy pancreatic tissue was predominantly localized in the acinar cells of the pancreas. The most frequently expressed biomarkers were integrin α_v_β_6_ and cMET that were both expressed in 88 % of the pancreatic adenocarcinoma cases (Table 3). In addition, cMET was abundantly expressed in 96 % of the periampullary adenocarcinoma patients (Table 4). To evaluate the ability of potential tumor-specific molecular markers to distinguish between pancreatic adenocarcinoma and healthy pancreatic tissue, the mean immunohistochemical intensity scores of the biomarkers were compared between both tissue types. In pancreatic adenocarcinoma, the mean intensity score for integrin α_v_β_6_ (*p* < 0.001; *p* < 0.001), CEA (*p* < 0.001; *p* < 0.001), EGFR (*p* < 0.001; *p* < 0.001), and uPAR (*p* < 0.001; *p* = 0.056) was significantly higher compared to normal pancreatic tissue (Fig. 1). In periampullary adenocarcinoma, the mean integrin α_v_β_6_ (*p* < 0.001), CEA (*p* < 0.001), and VEGFR2 (*p* = 0.045) staining intensity were significantly higher.

**Table 3:** Target Selection Criteria (TASC) score for integrin α_v_β_6_, carcinoembryonic antigen (CEA), hepatocyte growth factor receptor (cMET), epithelial growth factor receptor (EGFR), epithelial cell adhesion molecule (EpCAM), human epidermal growth factor receptor (HER2), urokinase receptor (uPAR), and vascular endothelial growth factor receptor 2 (VEGFR2) in pancreatic adenocarcinoma

Target	Extracellular localization of the protein (points awarded)	Pattern of upregulation (points awarded)	T/N ratio (points awarded)	Percentage with positive expression (points awarded)	Previous imaging success (points awarded)	Enzymatic activity (points awarded)	Internalization (points awarded)	TASC Score
Integrin α_v_β_6_	Membrane-bound (3) [99]	Diffuse (4)	Yes (3)	88 % (5)	Animal experiment (2) [68]	No (0) [100]	Yes (1) [65]	20
CEA	Membrane-bound (3) [101]	Diffuse (4)	Yes (3)	71 % (5)	Animal experiment (2) [74, 77]	Unknown (0)	Yes (1) [102]	20
UPAR	Membrane-bound (3) [71]	Diffuse (4)	Yes (3)	67% (3)	Animal experiment (2) [91]	Yes (1) [103]	Yes (1) [104]	19
cMET	Membrane-bound (3) [105]	Diffuse (4)	No (0)	88% (5)	Animal experiment (2) [106]	Yes (1) [107]	Yes (1) [108]	18
EGFR	Membrane-bound (3) [109]	Diffuse (4)	Yes (3)	69% (3)	In patients (2) [86, 110]	Unknown (0)	Yes (1) [111]	18
HER2	Membrane-bound (3) [112]	Diffuse (4)	No (0)	80% (5)	Animal experiment (2) [89]	Unknown (0)	Yes (1) [113]	17
VEGFR2	Membrane-bound (3) [114]	Focal (0)	No (0)	72% (5)	Animal experiment (2) [115]	Yes (1) [116]	Yes (1) [117]	14
EpCAM	Membrane-bound (3) [118]	Focal (0)	No (0)	59% (3)	Animal experiment (2) [119]	Unknown (0)	Yes (1) [120]	11

Extracellular localization of the protein was based on the literature; pattern of upregulation was obtained from the immunohistochemical staining (diffuse, staining in ≥50 % of tumor cells in the majority (>50 %) of the patients; focal, staining in <50 % of tumor cells in the majority (>50 %) of the patients; or negative, staining in 0 % of the tumor cells in the majority (>50 %) of the patients) described in this study; tumor to normal (T/N) ratio of the biomarker expression, as determined by significant higher mean immunohistochemistry staining (staining intensity was classified for every patient as follows: 0 = negative, 1 = weak, 2 = moderate, and 3 = strong) in pancreatic adenocarcinoma compared to normal pancreatic tissue; percentage of patients with positive expression were described according the findings of the current study; previous imaging success was defined as published *in vivo* tumor-specific imaging studies directed at the target; enzymatic activity refers to enzymatic activity of the target in and around the tumor described in the literature, that potentially can be used for locally activated probes; internalization indicates the receptor could have the ability to internalize the probe-target complex in the tumor cell according to previous studies

**Table 4:** Target Selection Criteria (TASC) score for integrin α_v_β_6_, carcinoembryonic antigen (CEA), hepatocyte growth factor receptor (cMET), epithelial growth factor receptor (EGFR), epithelial cell adhesion molecule (EpCAM), human epidermal growth factor receptor (HER2), urokinase receptor (uPAR), and vascular endothelial growth factor receptor 2 (VEGFR2) in periampullary adenocarcinoma

Target	Extracellular protein localization of the protein (points awarded)	Pattern of upregulation (points awarded)	T/N ratio (points awarded)	Percentage with positive expression (points awarded)	Previously imaged (points awarded)	Enzymatic activity (points awarded)	internalization (points awarded)	TASC Score
VEGFR2	Membrane-bound (3) [114]	Diffuse (4)	Yes (3)	86% (5)	Animal experiment (2) [115]	Yes (1) [116]	Yes (1) [117]	21
CEA	Membrane-bound (3) [101]	Diffuse (4)	Yes (3)	89% (5)	Animal experiment (2) [74, 77]	Unknown (0)	Yes (1) [102]	20
cMET	Membrane-bound (3) [105]	Diffuse (4)	No (0)	96% (6)	Animal experiment (2) [106]	Yes (1) [107]	Yes (1) [108]	19
EGFR	Membrane-bound (3) [109]	Diffuse (4)	Yes (3)	59% (3)	In patients (0) [86, 110]	Unknown (0)	Yes (1) [111]	18
Integrin α_v_β_6_	Membrane-bound (3) [99]	Diffuse (4)	Yes (3)	68% (3)	Animal experiment (2) [68]	No (0) [100]	Yes (1) [65]	18
EpCAM	Membrane-bound (3) [118]	Diffuse (4)	No (0)	68% (3)	Animal experiment (2) [119]	Unknown (0	Yes (1) [120]	17
HER2	Membrane-bound (3) [112]	Diffuse (4)	No (0)	88% (5)	Animal experiment (2) [89]	Unknown (0	Yes (1) [113]	17
UPAR	Membrane-bound (3) [71]	Focal (0)	Yes (3)	4% (0)	Animal experiment (2) [91]	Yes (1) [103]	Yes (1) [104]	12

Extracellular localization of the protein was based on the literature; pattern of upregulation was obtained from the immunohistochemical staining (diffuse, staining in ≥50 % of tumor cells in the majority (>50 %) of the patients; focal, staining in <50 % of tumor cells in the majority (>50 %) of the patients; or negative, staining in 0 % of the tumor cells in the majority (>50 %) of the patients) described in this study; tumor to normal (T/N) ratio of the biomarker expression, as determined by significant higher mean immunohistochemistry staining (staining intensity was classified for every patient as follows: 0 = negative, 1 = weak, 2 = moderate, and 3 = strong) in periampullary adenocarcinoma compared to normal pancreatic tissue; percentage of patients with positive expression; percentage of patients with positive expression were described according the findings of the current study; previous imaging success was defined as published *in vivo* tumor-specific imaging studies directed at the target; enzymatic activity refers to enzymatic activity of the target in and around the tumor described in the literature, that potentially can be used for locally activated probes; internalization indicates the receptor could have the ability to internalize the probe-target complex in the tumor cell according to previous studies

### Biomarker Panels

The combined expression of two biomarkers was evaluated to assess their potential as a dual target for tumor-specific imaging (Table 5). In pancreatic adenocarcinoma, integrin α_v_β_6_ and/or CEA were expressed in 99 % of the patients and 64 % of the cases expressed both integrin α_v_β_6_ and CEA, suggesting that the combination of both targets would be a promising approach for tumor-specific imaging. In periampullary adenocarcinoma, the most promising combination was CEA and EGFR, whereas all cases expressed either CEA and/or EGFR. In addition, integrin α_v_β_6_ and/or CEA were expressed in 96 % of the cases.

**Table 5:** Expression, as determined by immunohistochemistry, of biomarkers panels (combining the expression of two molecular markers) consisting of integrin α_v_β_6_, carcinoembryonic antigen (CEA), epithelial growth factor receptor (EGFR), and/or urokinase receptor (uPAR) in pancreatic and periampullary adenocarcinoma

Biomarker panel		Total population		Pancreatic adenocarcinoma		Periampullary adenocarcinoma	
		Overlapping expression	Total expression	Overlapping expression	Total expression	Overlapping expression	Total expression
Integrin α_v_β_6_	CEA	64 %	97 %	64 %	99 %	63 %	96 %
Integrin α_v_β_6_	uPAR	52 %	90 %	62 %	96 %	4 %	73 %
Integrin α_v_β_6_	EGFR	62 %	91 %	66 %	94 %	44 %	82 %
CEA	uPAR	43 %	91 %	50 %	91 %	4 %	88 %
CEA	EGFR	52 %	91 %	52 %	90 %	54 %	100 %
uPAR	EGFR	40 %	83 %	48 %	88 %	60 %	68 %

Overlapping expression refers to the percentage of patients that show positive expression (positive expression was defined as positive if >10 % of the tumor cells expressed a moderate or strong staining pattern) for both molecular markers in the biomarker panel. Total expression describes the frequency of patients that show positive expression (positive expression was defined as positive if >10 % of the tumor cells expressed a moderate or strong staining pattern) of one or both molecular markers in the biomarker panel and therefore could be visualized with a dual-tracer targeting both biomarkers

### TASC Score

The TASC score was calculated for all molecular markers evaluated in this study (Tables 3 and 4). Integrin α_v_β_6_ (20 points), CEA (20 points), uPAR (19 points), cMET (18 points), and EGFR (18 points) were considered suitable targets for tumor-specific imaging of pancreatic adenocarcinoma according the TASC score. For tumor-specific imaging of periampullary adenocarcinoma, VEGFR2 (21 points), CEA (20 points), cMET (19 points), EGFR (18 points), and integrin α_v_β_6_ (18 points) were categorized as potential targets by the TASC scoring system.

## Discussion

Tumor-specific intraoperative imaging is a rapidly emerging field that holds great promise to reduce tumor-positive resection margin rates in oncologic pancreatic surgery [30]. However, to make the transition to clinical practice, tumor-specific imaging targets and accompanying contrast agents are prerequisite [15]. Therefore, the present study strives to provide the first steps toward clinical translation by investigating the suitability of a set of molecular markers as potential targets for tumor-specific imaging of pancreatic adenocarcinoma. The results of this study show that integrin α_v_β_6_, CEA, EGFR, and uPAR are significantly upregulated in pancreatic adenocarcinoma compared to healthy pancreatic tissue and suggest that these biomarkers are promising targets for tumor-specific contrast agent development. By combining individual biomarkers in dual biomarker panels, the coverage of patients was increased: in pancreatic adenocarcinoma, considering almost the complete population expressed either integrin α_v_β_6_ and/or CEA. Furthermore, the TASC score confirmed the potential of integrin α_v_β_6_, CEA, EGFR, and uPAR as suitable targets for tumor-specific imaging.

Previous reports regarding the expression of integrin α_v_β_6_ (85–100 %), cMET (82–100 %), EGFR (36–69 %), EpCAM (56–78 %), HER2 (16–69 %), and VEGFR2 (64–93 %) in pancreatic adenocarcinoma are consistent with our results [31–47]. Preceding findings demonstrate a higher expression of CEA (98–100 %) and uPAR (90–96 %) in pancreatic adenocarcinoma to our findings; however, this slight discrepancy is not likely to alter the final conclusion of this study [33, 35, 48]. Furthermore, studies of others showed analogue to our results that cMET, EpCAM, and HER2 are overexpressed in healthy pancreatic tissue, which would render them less preferable as imaging targets [37, 38, 40–42]. Importantly, the expression of integrin α_v_β_6_, CEA, and uPAR has been described previously in compliance with our results, as very low or undetectable in normal pancreatic tissue, which would translate to a favorable tumor-to-background ratio when used for imaging purposes [31, 35, 48, 49]. EGFR and VEGFR2 were previously shown as respectively present and absent in normal pancreatic tissue, contradicting our findings [41, 42, 49]. This ambiguity highlights the need to further investigate the ability of EGFR and VEGFR to distinguish between normal and malignant pancreatic tissue, especially since fluorescence-labeled contrast agents directed at EGFR and VEGF, including bevacizumab-IRDye800CW, cetuximab-IRDye800CW, and panitumab-IRDye800CW, are in various stages of clinical trials for clinical use in several other types of cancer [15].

The results of this study are posed by limitations inherent to immunohistochemical analysis, such as variation in the quality of the primary antibodies, immunohistochemical staining techniques, scoring criteria, paraffin impregnation, surgical specimen fixation delay, or diversity in the ethnic distribution of the study population [50, 51]. In addition, the immunohistochemistry procedure, including tissue fixation and antigen retrieval, destroys the membrane integrity and protein conformation, which makes the protein less representative for its naive counterpart. The antibodies used in this study were not specifically selected for the development of tumor-specific probes, since the focus of this study was to identify the most suitable targets; however, the antibodies in this study used for integrin α_v_β_6_ (6.2A, Biogen Idec MA Inc.), CEA (A0155, Dako), EGFR (E30, Dako), EpCAM (323A3), and uPAR (ATN-615) react on the extracellular epitopes of their analogues and have been described for use on intact protein [52]. The latter could be promising for use in imaging probes. Furthermore, the normal pancreatic tissue used in this study was obtained in proximity of the tumor for an optimal representation of the reality of image-guided surgery. Premalignant biological changes may already exist in this presumed normal pancreatic tissue, which could explain for the differences between our findings and the biomarker expression in normal pancreatic tissue reported in the literature. For the purpose of this study, the term periampullary adenocarcinoma was used as an omnibus term for a very a heterogeneous group of adenocarcinoma that invade the head of the pancreas with distinctively different histology and expression of molecular markers as they originate from the duodenum, papilla of Vateri or the common bile duct. Hence, it is challenging or even impossible to draw conclusions that are true for the whole cohort periampullary adenocarcinoma based on our findings or represent them with a histology slide in Fig. 1. Moreover, this study applies a threshold of over 10 % medium or dark stained tumor cells on 2-mm core TMAs to define tumor positivity. Therefore, the results of this study do not provide conclusive evidence on whether the evaluated targets could be used for tumor-specific imaging of the complete tumor and all residual disease. Nevertheless, the results of this study provide guidance on which molecular makers show the most promise for further investigation as tumor-specific imaging targets. Likewise, the reported expression of the composed biomarker panels investigated in this study indicates which biomarker combinations show complementary instead of overlapping expression in the majority of pancreatic adenocarcinoma and subsequently holds promise for future more elaborate examination. However, considering the >10 % threshold, these results are not decisive on whether dual tracers directed at the inquired biomarker panels will be able to visualize the entire disease burden.

The TASC score identified cMET as a promising imaging target for pancreatic adenocarcinoma, whereas cMET did not significantly differentiate between healthy and malignant pancreatic tissue in our hands. These results suggest that the TASC score still experiences teething trouble and needs further validation and adaptation, since distinguishing between normal and malignant tissue is considered the cornerstone of surgical oncology. Various therapeutic antibodies have been investigated in preclinical models for imaging of cancer, including cetuximab, panitumumab, and bevacizumab [53–56]. Human clinical trials are underway, but none of these biologics are presently available for intraoperative imaging in humans. Use of an FDA-approved targeting molecule facilitates clinical translation, because it lowers the cost barrier to clinical practice, since revenue associated with diagnostic agents is significantly lower than for therapeutic agents [16, 57]. Therefore, for future use, the TASC score should also take into consideration the availability of FDA-approved antibodies. Nevertheless, de novo development of intraoperative diagnostics also takes place, for example, the Arg-Gly-Asp (RGD) peptide has a high affinity and selectivity for multiple integrins, among them integrin α_v_β_6_, and has extensively been studied for imaging objectives [58, 59]. In addition, another example of *de novo* developed imaging probes are autoquenched fluorescent probes, such as ProSense, that convert from a nonfluorescent to fluorescent state by proteolytic activation of lysosomal cysteine or serine proteases, hence the value of including enzymatic activity in by the TASC score [60]. Furthermore, the TASC criteria could be elaborated by adding points to the score for targets with a soluble form that can be targeted by certain antibodies, such as the ATN-615 antibody that recognizes a soluble form of uPA in addition to uPAR, which allows for antibodies to also target receptors that are already occupied by its soluble form thereby increasing its reach. Nevertheless, the TASC score is a promising tool to incorporate other favorable characteristics of potential imaging targets for pancreatic adenocarcinoma in a weighted and standardized manner in our judgment.

Despite the previously mentioned limitations, this study was to the best of our knowledge the first study to assess the ability of potential targets for the image-guided surgery of pancreatic adenocarcinoma to distinguish between normal and malignant pancreatic tissue in a relatively large cohort of patients with pancreatic adenocarcinoma using the TASC score. In addition, this study was also able to investigate the expression of potential imaging targets in periampullary adenocarcinoma. The latter is of added value since the histological origin of pancreatic head masses is often unknown in wait of pancreatic surgery. Furthermore, this study was to our knowledge the first to describe the combined expression of potential imaging targets to facilitate future development of dual-labeled imaging probes; however, these dual-purpose agents present additional hurdles in development and clinical translation that are beyond the scope of this article before their potential is fully realized [16]. Moreover, aside from providing guidance for tumor surgery, molecular imaging techniques also play an increasingly important role in the preoperative staging and guidance of cancer therapy in pancreatic adenocarcinoma patients [61].

In conclusion, tumor-targeted intraoperative imaging of pancreatic adenocarcinoma has great potential to improve pancreatic surgery [12, 62]. However, the clinical implementation of this novel technique is currently halted by the lack of clinically approved tumor-specific contrast agents. Therefore, the present study sought to pave the way for future development of tumor-specific contrast agents and consecutive image-guided resection of pancreatic adenocarcinoma, by investigating the most suitable molecular targets for tumor-specific imaging. The results of this study show that a dual-targeted tracer aimed at both integrin α_v_β_6_ and CEA would be able to detect tumor cells in 99 % of all pancreatic cancer patients.
